# The Mediator Complex Subunits MED14, MED15, and MED16 Are Involved in Defense Signaling Crosstalk in *Arabidopsis*

**DOI:** 10.3389/fpls.2016.01947

**Published:** 2016-12-22

**Authors:** Chenggang Wang, Xuezhu Du, Zhonglin Mou

**Affiliations:** ^1^Department of Microbiology and Cell Science, University of Florida, GainesvilleFL, USA; ^2^College of Life Science, Hubei UniversityWuhan, China

**Keywords:** MED14, MED15, MED16, salicylic acid, jasmonate, and ethylene, defense signaling crosstalk, *Arabidopsis*

## Abstract

Mediator is a highly conserved protein complex that functions as a transcriptional coactivator in RNA polymerase II (RNAPII)-mediated transcription. The *Arabidopsis* Mediator complex has recently been implicated in plant immune responses. Here, we compared salicylic acid (SA)-, methyl jasmonate (MeJA)-, and the ethylene (ET) precursor 1-aminocyclopropane-1-carboxylic acid (ACC)-induced defense and/or wound-responsive gene expression in 14 *Arabidopsis* Mediator subunit mutants. Our results show that MED14, MED15, and MED16 are required for SA-activated expression of the defense marker gene *PATHOEGNESIS-RELATED GENE1*, MED25 is required for MeJA-induced expression of the wound-responsive marker gene *VEGATATIVE STORAGE PROTEIN1* (*VSP1*), MED8, MED14, MED15, MED16, MED18, MED20a, MED25, MED31, and MED33A/B (MED33a and MED33B) are required for MeJA-induced expression of the defense maker gene *PLANT DEFENSIN1.2* (*PDF1.2*), and MED8, MED14, MED15, MED16, MED25, and MED33A/B are also required for ACC-triggered expression of *PDF1.2*. Furthermore, we investigated the involvement of MED14, MED15, and MED16 in plant defense signaling crosstalk and found that MED14, MED15, and MED16 are required for SA- and ET-mediated suppression of MeJA-induced *VSP1* expression. This result suggests that MED14, MED15, and MED16 not only relay defense signaling from the SA and JA/ET defense pathways to the RNAPII transcription machinery, but also fine-tune defense signaling crosstalk. Finally, we show that MED33A/B contributes to the necrotrophic fungal pathogen *Botrytis cinerea-*induced expression of the defense genes *PDF1.2, HEVEIN-LIKE*, and *BASIC CHITINASE* and is required for full-scale basal resistance to *B. cinerea*, demonstrating a positive role for MED33 in plant immunity against necrotrophic fungal pathogens.

## Background

Salicylic acid (SA), jasmonates (JAs), and ethylene (ET) are the primary defense signal molecules of the plant immune system (Pieterse et al., 2009). SA activates resistance against biotrophs and hemibiotrophs, JA mediates defense against necrotrophs and responses to wounding and herbivores, and ET contributes to defense signaling against necrotrophs (Pozo et al., 2004; van Loon et al., 2006; Loake and Grant, 2007). While each of these signal molecules induces a specific defense signaling pathway(s), there is extensive crosstalk among them (Thomma et al., 2001; Glazebrook, 2005; Pieterse et al., 2009). For instance, SA and JA signaling mostly antagonize each other, and ET enhances both SA- and JA-mediated defense responses against pathogens, but suppresses JA-mediated wound signaling. Such crosstalk allows plants to prioritize one defense response over others when encountering a specific attacker. Defense signaling crosstalk has been extensively studied in recent years (Pieterse et al., 2009), but the underlying molecular mechanisms still await full characterization.

Mediator is a highly conserved protein complex that is essential for RNA polymerase II (RNAPII)-mediated transcription (Conaway and Conaway, 2011). This protein complex exists in multiple functionally distinct forms and acts as either a transcriptional activator or a repressor, depending on its associated protein partners. The Mediator core contains more than 20 subunits, which are organized into head, middle, and tail modules (Guglielmi et al., 2004; Chadick and Asturias, 2005). Mediator associates with the RNAPII complex via the head and middle modules to form the holoenzyme, which stimulates basal transcription and supports activation of transcription by specific transcriptional activators (Ansari et al., 2009). By interacting with particular transcriptional activators, individual Mediator subunits converge diverse signals to the RNAPII transcription complex, leading to pathway-specific gene transcription (Balamotis et al., 2009). The head and middle modules of Mediator can also interact with a kinase module, which prevents their binding to the RNAPII complex, leading to transcriptional repression (Knuesel et al., 2009). The *Arabidopsis* Mediator complex contains 27 conserved subunits and six additional subunits whose positions in the complex are unassigned (Bäckström et al., 2007; Mathur et al., 2011). A number of the *Arabidopsis* Mediator subunits have been implicated in immune responses. For instance, MED14, MED15, MED16, and MED19a have been shown to regulate the SA-triggered immunity against biotrophic and hemibiotrophic pathogens (Canet et al., 2012; Zhang et al., 2012, 2013; Caillaud et al., 2013), whereas MED8, MED12, MED13, MED14, MED16, MED21, MED25, and CDK8 have been found to function in JA/ET-mediated immunity against necrotrophic pathogens (Dhawan et al., 2009; Kidd et al., 2009; Zhang et al., 2012; Zhu et al., 2014). MED18 also functions in resistance to necrotrophic pathogens, but the resistance appears to be independent of the JA/ET signaling (Lai et al., 2014).

We have previously shown that the *Arabidopsis* Mediator complex subunit MED16 is required for ET-promoted inhibition of JA-mediated wound signaling (Wang et al., 2015b), indicating that some of the Mediator subunits may be involved in defense signaling crosstalk. Here, we compared SA-, methyl jasmonate (MeJA)-, and the ET precursor 1-aminocyclopropane-1-carboxylic acid (ACC)-induced defense and/or wound-responsive marker gene expression in 14 *Arabidopsis* Mediator subunit mutants and identified MED14, MED15, and MED16 as key players in plant defense signaling crosstalk. Additionally, we found that the Mediator subunits MED33A and MED33B (MED33A/B) positively contribute to *Arabidopsis* defense responses against the necrotrophic fungal pathogen *Botrytis cinerea*.

## Materials and Methods

### Plant Materials and Growth Conditions

The wild-type used in this study was the *Arabidopsis thaliana* (L.) Heynh. ecotype Columbia (Col-0). All Mediator mutants except *med33b* were previously described (Wang et al., 2015b). The *med33b* mutant (SALK_037472) and *med15*/*nbr4-4* (SAIL_792_F02) were obtained from the *Arabidopsis* Biological Resource Center at The Ohio State University (Columbus, OH, USA). Homozygous mutant plants of SALK_037472 were confirmed with primers (forward: 5′GTACGAGGTTGCAACTACTG3′and reverse: 5′GCAGTGGAGAAAACAGCATG3′) that flank the T-DNA insertion and the left border primer LBa1 (Alonso et al., 2003). The *med33a/b* double mutant was created by crossing SALK_037472 with SALK_022477 (*med33a*) and identified in the F_2_ generation by PCR. The *Arabidopsis* seeds were sown on autoclaved soil (Sunshine MVP; Sun Gro Horticulture, Agawam, MA, USA) and cold-treated at 4°C for 3 days. Plants were germinated and grown at 22–24°C under a 16-hr-light/8-hr-dark regime. Four-week-old soil-grown plants were used for pathogen infection.

### Chemical Treatment

Ten-day-old seedlings grown on one-half-strength Murashige and Skoog (½12 × MS) medium were treated with 0.5 mM SA, 0.1 mM MeJA, 0.1 mM ACC, or their combination. Seedlings for the negative control were treated with water. Aerial parts of the seedlings were collected and subjected to total RNA extraction.

### Pathogen Infection

The *B. cinerea* strain B05 was used in this study. *B. cinerea* inoculation and lesion size measurement were conducted as described in detail previously (Wang et al., 2015a).

### RNA Analysis

Total RNA extraction, reverse transcription, and real-time quantitative PCR (qPCR) were performed as previously described (Defraia et al., 2010). Primers used for *PR1, VSP1*, and *PDF1.2* were described previously (Defraia et al., 2010; Wang et al., 2015b). Primers used for *HEL* are forward: 5′GTGAGTGCTTATTGCTCCAC3′ and reverse: 5′ACATCCAAATCCAAGCCTCC3′, and for *CHIB* are forward: 5′GGTTCTGGATGACTGCTCAG3′ and reverse: 5′CTATACGATCGGCGACTCTC3′.

### Statistical Methods

Statistical analyses were performed using the one-way ANOVA and the two-way ANOVA in Prism 5.0b (GraphPad Software, La Jolla, CA, USA). Lesion sizes measured in three independent experiments were combined and analyzed as a one-way ANOVA, blocked by experiment, using JMP 11 (JMP Software, Cary, NC, USA). All experiments were repeated three independent times with similar trends. Results from a representative experiment are presented.

## Results

### SA-, MeJA, and ACC-Induced Defense Marker Gene Expression in 14 Mediator Mutants

**FIGURE 1:**
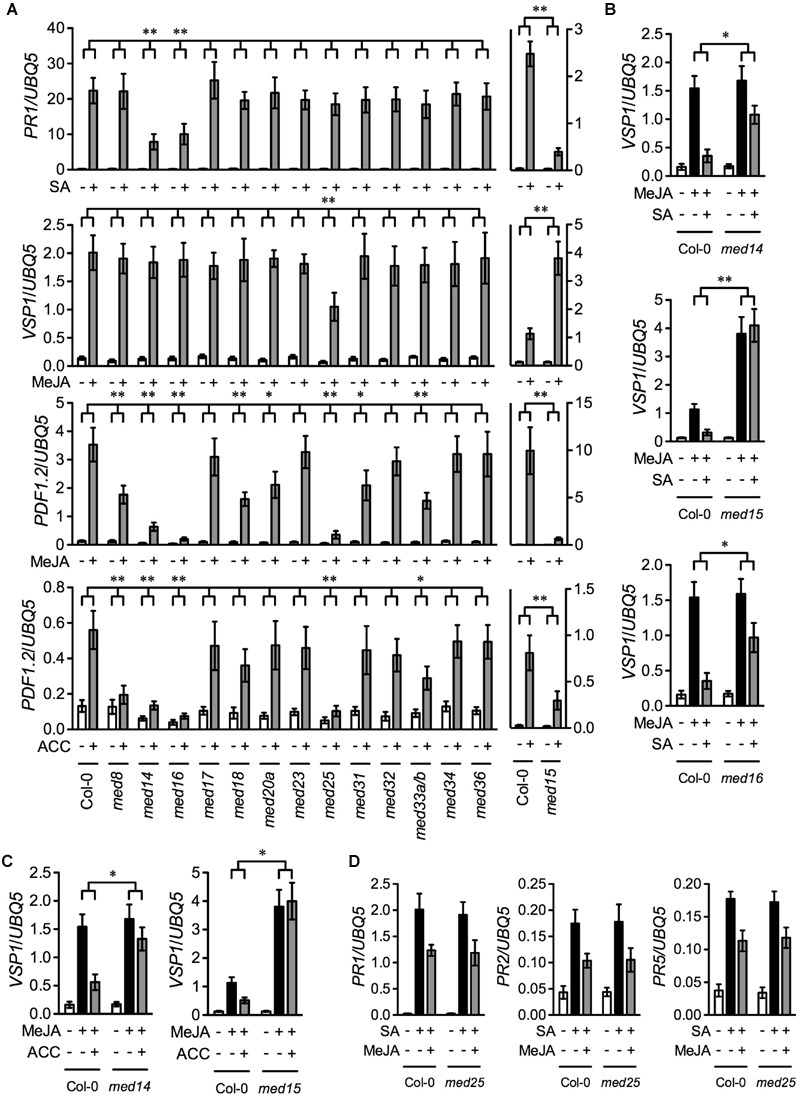
**SA-, MeJA-, ACC-, and their combinations-induced pathogen- and wound-responsive genes in Mediator subunit mutants. (A)** SA-induced *PR1*, MeJA-induced *VSP1* and *PDF1.2*, and ACC-induced *PDF1.2* in 14 Mediator subunit mutants. Ten-day-old seedlings of Col-0 and the indicated Mediator mutants except *med15* grown on ½12 × MS medium as well as 3-week-old soil-grown Col-0 and 7-week-old soil-grown *med15* plants were treated with 0.5 mM SA, 0.1 mM MeJA, or 0.1 mM ACC. Plant tissues were collected 6 h after the treatment for analysis of *VSP1* and 24 h for *PR1* and *PDF1.2*. **(B)** SA-mediated inhibition of MeJA-induced expression of *VSP1* in *med14, med15*, and *med16*. Ten-day-old Col-0, *med14*, and *med16* seedlings grown on ½12 × MS medium as well as 3-week-old soil-grown Col-0 and 7-week-old soil-grown *med15* plants were treated with 0.1 mM MeJA or 0.1 mM MeJA plus 0.5 mM SA. Plant tissues were collected 6 h after the treatment. **(C)** ET-mediated inhibition of MeJA-induced expression of *VSP1* in *med14* and *med15*. Ten-day-old Col-0 and *med14* seedlings grown on ½12 × MS medium as well as 3-week-old soil-grown Col-0 and 7-week-old soil-grown *med15* plants were treated with 0.1 mM MeJA or 0.1 mM MeJA plus 0.1 mM ACC. Plant tissues were collected 6 h after the treatment. **(D)** SA-induced *PR* gene expression in *med25* in the presence and absence of MeJA. Ten-day-old Col-0 and *med25* seedlings grown on ½12 × MS medium were treated with 0.5 mM SA or 0.5 mM SA plus 0.1 mM MeJA. Plant tissues were collected 24 h after the treatment. Total RNA was extracted from the collected plant tissues and subjected to real-time qPCR analysis. Expression of the target genes was normalized against the constitutively expressed *UBQ5*. Data represent means of three biological replicates with standard deviation (SD). Asterisks indicate that the induction of the gene was significantly lower or higher **(A)** and the inhibition of MeJA-induced *VSP1* expression was significantly weaker **(B,C)** in the mutant than in the Col-0 plants (^∗^*P* < 0.05, ^∗∗^*P* < 0.01, two-way ANOVA).

To compare the function of different *Arabidopsis* Mediator subunits in the SA, JA, and ET signaling pathways, we tested SA-induced expression of the SA pathway marker gene *PATHOEGNESIS-RELATED GENE1* (*PR1*), MeJA-induced expression of the wound-responsive marker gene *VEGATATIVE STORAGE PROTEIN1* (*VSP1*) and the defense marker gene *PLANT DEFENSIN1.2* (*PDF1.2*), and ACC-induced expression of *PDF1.2* in the previously described 13 Mediator subunit mutants except that a *med33a/b* double mutant was used to replace the *med33b* single mutant (Wang et al., 2015b). Ten-day-old seedlings of the wild-type Col-0 and the 13 Mediator mutants grown on ½12 × MS medium were treated with SA, MeJA, or ACC. We also included the *med15*/*nrb4-4* mutant in the experiment, as MED15 is essential for SA signaling (Canet et al., 2012). Since homozygous *med15* plants are sterile, we used seeds from heterozygous plants. Three weeks after germination in soil, the small and chlorotic homozygous *med15* plants were transplanted and allowed to grow for four more weeks. As *med15* mutant plants grow very slowly compared with Col-0, 3-week-old soil-grown Col-0 plants with a size similar to that of the *med15* plants were used for comparison. As shown **Figure 1A**, SA-induced *PR1* expression was significantly blocked in *med14, med15*, and *med16*, MeJA-induced *VSP1* expression was significantly reduced only in *med25*, MeJA-induced *PDF1.2* expression was significantly decreased in *med8, med14, med15, med16, med18, med20a, med25, med31*, and *med33a/b*, and ACC-induced *PDF1.2* expression was significantly inhibited in *med8, med14, med15, med16, med25*, and *med33a/b*. Note that the observation that MeJA-induced *PDF1.2* expression was significantly decreased in *med18* is in contrast to the previous report (Lai et al., 2014). This discrepancy is probably due to different growth conditions. Nevertheless, these results indicate that, among the 14 Mediator subunits, MED14, MED15, and MED16 are required for SA-activated *PR1* expression, MED25 is required for MeJA-induced *VSP1* expression, MED8, MED14, MED15, MED16, MED18, MED20a, MED25, MED31, and MED33A/B are required for MeJA-induced *PDF1.2* expression, and MED8, MED14, MED15, MED16, MED25, and MED33A/B are required for ACC-induced *PDF1.2* expression.

### Involvement of MED14, MED15, and MED16 in Defense Signaling Crosstalk

Since MED14, MED15, and MED16 function in the SA pathway (Canet et al., 2012; Wathugala et al., 2012; Zhang et al., 2012, 2013), they might also be involved in SA-mediated suppression of JA signaling. To test this hypothesis, we treated *med14, med15, med16*, and Col-0 plants with MeJA or MeJA plus SA and examined the induction of the MeJA-induced wound-responsive gene *VSP1*. As shown in **Figure 1B**, SA significantly inhibited MeJA-induced expression of *VSP1* in the Col-0 plants, but the inhibition was significantly alleviated in *med14* and *med16*, and completely blocked in *med15*, indicating that MED14, MED15, and MED16 are all required for SA-mediated suppression of JA-mediated wound-responsive gene expression. Moreover, MED14, MED15, and MED16 also function in the ET-mediated defense pathway. As MED16 is required for ET-activated suppression of JA-mediated wound signaling (Wang et al., 2015b), MED14 and MED15 might also be required for this process. To test this, we treated *med14, med15*, and Col-0 plants with MeJA or MeJA plus ACC and tested the induction of *VSP1*. As shown in **Figure 1C**, ACC significantly inhibited MeJA-induced expression of *VSP1* in the Col-0 plants, but the inhibition was dramatically relieved in *med14* and completely blocked in *med15*. Therefore, as MED16 (Wang et al., 2015b), MED14 and MED15 are also required for ET-mediated suppression of JA-induced wound-responsive gene expression. Finally, since MED25 functions in JA-mediated pathogen and wound responses (Kidd et al., 2009; Chen et al., 2012), it might modulate JA-mediated suppression of SA signaling. To test this hypothesis, we treated *med25* and Col-0 plants with SA and SA plus MeJA and examined the induction of the SA-responsive genes *PR1, PR2*, and *PR5*. As shown in **Figure 1D**, MeJA inhibited SA-induced *PR* gene expression to similar extents in the Col-0 and *med25* plants, indicating that MED25 is not involved in JA-mediated suppression of SA signaling.

### Function of MED33A/B in Basal Resistance against the Necrotrophic Fungal Pathogen *B. cinerea*

**FIGURE 2:**
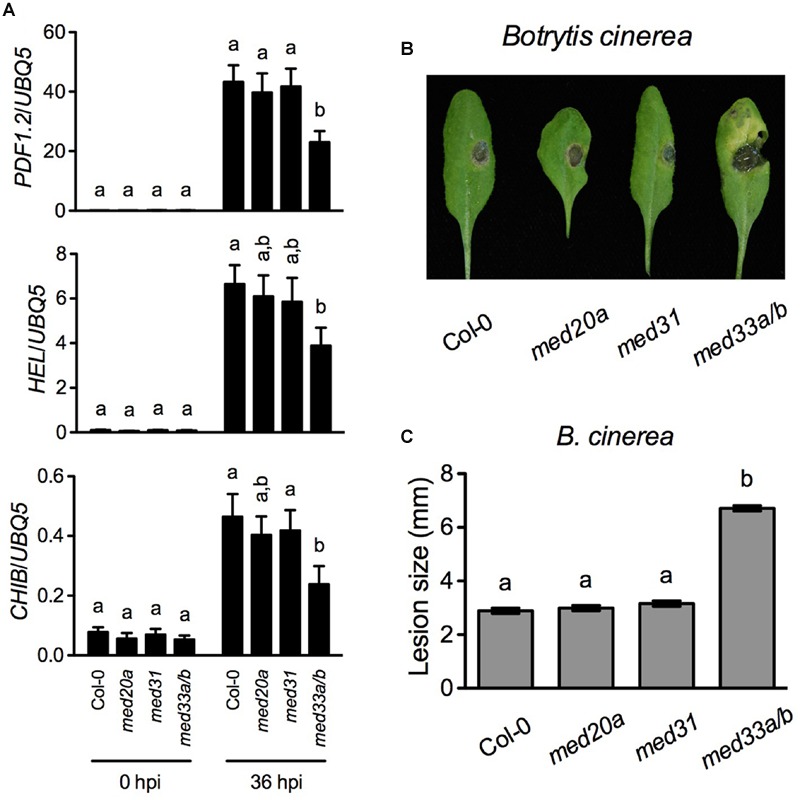
***Botrytis cinerea*-induced defense responses in *med20a, med31*, and *med33a/b.* (A)** *B. cinerea*-induced expression of *PDF.2, HEL*, and *CHIB* in Col-0, *med20a, med31*, and *med33a/b* plants. Leaf tissues were collected 36 h post-inoculation (hpi). RNA extraction and real-time qPCR were performed as in **Figure 1**. Data represent means of three biological replicates with SD. Different letters above the bars indicate significant differences (*P* < 0.05, on-way ANOVA). The statistical comparisons were performed among genotypes for each time point. **(B)** Disease symptoms on rosette leaves of 4-week-old soil-grown plants inoculated with *B. cinerea*. Photos were taken 4 days post-inoculation. **(C)** Size of the necrotrophic lesions formed on *B. cinerea*-infected Col-0, *med20a, med31*, and *med33a/b* plants. Lesion sizes on 90 leaves measured in three independent experiments were combined and analyzed as a one-way ANOVA, blocked by experiment. The resulting mean and standard error are presented. Different letters above the bars indicate significant differences (*P* < 0.0001).

*PDF1.2* is a marker gene of the JA/ET-mediated defense signaling, which is central in resistance to necrotrophic pathogens. The Mediator subunits MED8, MED14, MED16, MED18, and MED25 are required for MeJA- and/or ACC-induced *PDF1.2* expression and contribute to resistance to necrotrophic fungal pathogens (Kidd et al., 2009; Wathugala et al., 2012; Zhang et al., 2012; Lai et al., 2014; Wang et al., 2015b). Since MED20a, MED31, and MED33A/B are also required for full induction of *PDF1.2* by MeJA and/or ACC, we examined *B. cinerea*-induced expression of three JA/ET-responsive genes *PDF1.2, HEVEIN-LIKE* (*HEL*), and *BASIC CHITINASE* (*CHIB*) in *med20a, med31*, and *med33a*/*b* and tested resistance of these mutants to *B. cinerea*. We did not include the *med15* mutant in the experiment due to its extremely delayed growth. As shown in **Figure 2A**, *B. cinerea*-induced expression of *PDF1.2, HEL*, and *CHIB* was significantly reduced only in the *med33a/b* double mutant. Consistently, *med33a/b* also exhibited enhanced susceptible to *B. cinerea* (**Figures 2B,C**). These results indicate that MED33A/B plays a positive role in defense against this necrotrophic fungal pathogen.

## Discussion

It is generally accepted that the tail module of Mediator is the main target for transcriptional activators. The *Arabidopsis* Mediator tail module consists of MED14, MED15, MED16, MED23, MED27, MED32, and MED33 (Mathur et al., 2011). In this study, we characterized T-DNA insertion mutants of all tail module subunits except MED27, for which no homozygous T-DNA insertion line was identified. Our results show that, besides MED16 (Wathugala et al., 2012; Zhang et al., 2012), MED14, MED15, and MED33A/B are also required for full induction of the defense marker gene *PDF1.2* by MeJA and ACC (**Figure 1A**). MED33A/B also contributes to *B. cinerea-*induced defense gene expression and is required for full-scale basal resistance against this necrotrophic fungal pathogen (**Figures 2A–C**).

Importantly, we found that the tail module subunits MED14, MED15, and MED16 not only play dominant roles in regulation of both SA- and JA/ET-mediated defense responses (Canet et al., 2012; Wathugala et al., 2012; Zhang et al., 2012, 2013), but also are required for both SA- and ET-promoted inhibition of JA-mediated wound signaling (**Figures 1B,C**). These results indicate that MED14, MED15, and MED16 not only relay defense signaling from the SA and JA/ET pathways to the RNAPII transcription machinery, but also fine-tune defense-related transcriptional changes. We have recently shown that the transcription factor WRKY33, which is an important regulator of defense against necrotrophic fungal pathogens (Zheng et al., 2006), delivers signals to Mediator by interacting with MED16 (Wang et al., 2015b). The SA pathway transcriptional coactivator NONEXPRESSER OF PR GENE1 and TGA transcription factors as well as the JA/ET defense pathway transcription factors ETHYLENE INSENSITIVE3 (EIN3), ETHYLENE INSENSITIVE3-LIK1 (EIL1), OCTADECANOID-RESPONSIVE *ARABIDOPSIS* AP2/ERF59 (ORA59), and ETHYLENE RESPONSIVE FACTOR (ERF1) may also deliver signals to Mediator through the tail module. Indeed, it has been shown that EIN3, EIL1, ORA59, and ERF1 all interact with MED25, which in turn is physically associated with MED16 (Cevik et al., 2012; Yang et al., 2014). Whether any of the SA and JA/ET defense pathway transcriptional activators interact directly with MED14, MED15, and/or MED16 awaits further investigation.

## Author Contributions

ZM and CW conceived and designed the experiments. CW and XD performed the experiments. CW and ZM analyzed the data. ZM and CW wrote the paper. All of the authors carefully checked an approved this version of the manuscript.

## Conflict of Interest Statement

The authors declare that the research was conducted in the absence of any commercial or financial relationships that could be construed as a potential conflict of interest.
